# Personalized Physical Exercise Program Among Adolescent Girls: A Pilot Study

**DOI:** 10.3390/jfmk10030341

**Published:** 2025-09-06

**Authors:** Peter Petrovics, Balazs Sebesi, Zsolt Szekeres, Eszter Szabados, Anita Pálfi

**Affiliations:** 1 Institute of Sport Science and Physical Education, Faculty of Sciences, University of Pecs, H-7624 Pecs, Hungary; petropet@gamma.ttk.pte.hu (P.P.); sebesi.balazs@pte.hu (B.S.); 2 Department of Laboratory Medicine, Medical School, University of Pecs, H-7624 Pecs, Hungary; szekeres.zsolt@pte.hu; 3 Division of Preventive Cardiology and Rehabilitation, 1st Department of Medicine, Medical School, University of Pecs, H-7623 Pecs, Hungary

**Keywords:** personalized physical exercise, adolescent girls, cardiorespiratory performance, muscular strength, BMI, body composition

## Abstract

**Objectives:** Adolescence is a pivotal stage of development characterized by significant physical, psychological, and social changes. Establishing healthy lifestyle habits during this period is crucial for long-term health and the prevention of chronic diseases. Despite this, global trends show a marked decline in physical activity among adolescents, particularly girls, who are more susceptible to sedentary behaviors. One potential site for intervention to eliminate physical inactivity at the population level is the school educational setting during childhood. Traditional school-based physical exercise programs often adopt a one-size-fits-all approach, which may not address the diverse needs and interests of students, leading to reduced motivation and participation. Personalized physical exercise programs, tailored to individual capabilities and preferences, offer a promising alternative to enhance physical fitness and foster lifelong engagement in physical activity. **Methods:** A total of 170 Hungarian high school girls (mean age ≈ 15.3 years) were randomly assigned to either a personalized physical exercise group or a control group following the standard curriculum. The intervention spanned two academic years and consisted of five traditional gym classes per week (control group) or three traditional and two individually tailored classes with cardiorespiratory and resistance training per week (intervention group), each lasting 45–60 min. Individual goals were set based on baseline assessments, emphasizing self-referenced progress. **Results:** The personalized physical exercise group showed significant improvements in body mass index (BMI), body fat percentage, maximum oxygen uptake capacity (VO_2_max), muscular strength, and flexibility (*p* < 0.05), while the control group exhibited minimal or negative changes. **Conclusions:** The personalized physical exercise program has been shown to be more effective in achieving higher cardiorespiratory performance and favorable body composition among adolescent girls than a traditional school physical education class, highlighting its potential role in school settings.

## 1. Introduction

Adolescence is a critical period for both physical and psychosocial development [1]. Establishing healthy habits during this life stage can significantly impact quality of life and reduce the risk of chronic diseases later in life [2]. A physically active lifestyle has long been one of the cornerstones of adolescent health and development [3]. Regular physical activity not only improves physical fitness and cardiometabolic health, but also has a significant impact on mental well-being, academic performance, and the establishment of lifelong healthy habits [4,5]. However, recent research highlights a decline in physical activity levels among adolescents, with many failing to meet the recommended daily activity levels [6]. This trend is concerning, as sedentary behavior is a major contributor to the global burden of noncommunicable diseases (NCDs), such as obesity, type 2 diabetes, cardiovascular diseases, and certain types of cancer [7].

The World Health Organization’s (WHO) Global Physical Activity Action Plan 2018–2030 was developed through a worldwide consultation process involving governments and various sectors and stakeholders, such as health, sport, urban design, civil society, academia, and the private sector. Its aim is to reduce the global prevalence of physical inactivity in adolescents and adults by 15 percent by the year 2030, through the application of four strategic objectives: creating active societies, active environments, active people, and active systems [8]. One potential site for intervention to eliminate physical inactivity in adolescents is the school environment [9,10]. The objectives of physical education in schools are to encourage students to engage in health-improving physical activity and to teach them the knowledge and skills necessary for lifelong physical activity [9].

Numerous professional associations and medical societies, such as the American Cancer Society (ACS), American Diabetes Association (ADA), and American Heart Association (AHA), as well as government agencies, formally support the importance of physical activity for youth and the need for quality physical education in schools [11,12,13]. Characteristics of high-quality physical education include adequate time and facilities for physical education classes, a qualified physical education specialist delivering the program, providing as many practice opportunities as possible during classroom activities, and well-planned lessons that promote student learning [11].

Some studies have, however, found that the impact of compulsory physical exercise classes in schools is not enough to offset the adverse change in body composition and declining endurance in children who have developed as a result of a sedentary lifestyle in recent decades [14]. The reason why school physical education classes do not always achieve the desired results in increasing children’s cardiorespiratory fitness remains to be clarified. Although physical exercise is known to improve fitness parameters, traditional, one-size-fits-all school physical education programs often fail to address the diverse needs, preferences, and abilities of students [3]. Recently, attention has therefore turned towards personalized physical exercise training programs [15]. Unlike conventional physical education classes, which apply a uniform curriculum to all students, these programs consider students’ varying fitness levels, interests, and health conditions. Research results in adults comparing standardized and individualized training programs have already suggested that individualized training prescription methods using individual metabolic characteristics, such as ventilatory threshold, are more effective in improving cardiorespiratory fitness parameters than standardized approach using heart rate reserve [16].

Affective responses are also increasingly recognized as potentially effective intervention targets of individualized physical training methods that can promote physical activity and change physical activity-related behavior. It was shown that individualized exercise training could not only increase participation, but was also effective in improving fitness and strength [17,18].

The individualized approach not only enhances physical performance but also fosters intrinsic motivation, which is essential for sustaining physical activity beyond the school years [15]. Establishing appropriate goals and achieving early success can enhance effectiveness, build intrinsic motivation, and ultimately help maintain a physically active lifestyle [19].

When designing our study, we also considered the WHO’s observation that the physical activity of adolescent girls is significantly lower than expected, which constitutes a public health concern that calls for immediate and evidence-based policy action [20]. Boys generally perform better than girls in muscular strength, power, and endurance, and physical performance generally increases at a faster rate in boys during adolescence [13,21]. Previous studies have shown that adolescent girls are more prone to a sedentary lifestyle than boys. From a psychosocial perspective, they also showed lower levels of physical well-being, psychological well-being, and body satisfaction compared to boys [1,22]. It is also important to consider that girls’ needs and preferences differ even within school physical activity settings, as girls may prefer enjoyable, noncompetitive activities and value having the autonomy to decide which activities to participate in [20,23].

Considering the slower development of cardiorespiratory performance, the greater inclination to a sedentary lifestyle, and the psychological differences between boys and girls, it would be important to develop physical activity programs adapted to the interests and needs of adolescent girls, and that are both effective and enjoyable for them.

### Research Objectives

In the present study, we aimed to investigate the effects of a personalized physical exercise program on cardiorespiratory performance, muscle strength, BMI, and body composition among adolescent girls, and to compare the results with those of girls participating in traditional school physical education classes.

## 2. Materials and Methods

### 2.1. Subjects

A total of 170 high school girls voluntarily participated in the study, of whom 85 were randomly assigned to the experimental group and 85 to the control group. The mean age of the experimental group was 15.29 ± 0.5 years, while the control group had a mean age of 15.32 ± 0.51 years. Written informed consent was obtained from all the participating adolescents for the measurements and the anonymous use of data purely for scientific purposes. Parents were also asked to sign the form authorizing the measurements and data handling. The study was approved by the Regional Ethics Committee of the University of Pecs (7522-PTE 2018).

Inclusion criteria required participants to be high school students who were not involved in competitive sports during the study period and who consented to participate in the study. Exclusion criteria included medical conditions that prevented students from participating in physical activity classes and a lack of consent to participate in the study.

Both groups consisted of high school students who participated in regular, school-based physical education classes five times a week, each lasting 45–60 min. Their activities included sports such as running, gymnastics, and ball games, including soccer, basketball, handball, and volleyball. In the experimental group, two traditional physical education classes were replaced by personalized 45–60-minute sessions, held twice a week for two consecutive school years. Their activities included a structured regimen consisting of warm-up, main workout, and cool-down phases. The warm-up phase in the intervention group involved dynamic movements such as running in place and jumping. The main workout phase consisted of aerobic endurance or resistance elements in the form of circuit training, during which periods of intense activity were followed by rest intervals. The cool-down phase included stretching exercises. Individual goals were established for each adolescent based on their performance during baseline assessments. The training in the experimental group was conducted by a physical education teacher, who was a member of the research team. The exercises performed during the training were adapted to the students’ age and physical condition.

The specific exercises performed were categorized as follows:

Plyometrics: Explosive movements aimed at increasing lower-body strength. Examples include the following: Power Jumps: Raising knees during a jump and then returning to the starting position.

Cardio: High-intensity cardiovascular exercises aimed at fat burning. Examples include the following: High Knees: Rapid knee lifts while running in place. Burpees: Jumping from a squat to a push-up position, then returning and jumping up.

Core: Abdominal exercises aimed at strengthening and defining the core muscles. Examples include the following: Plank: Holding a push-up position with the body in a straight line. Bicycle Crunches: Alternating knee-to-elbow touches while lying down.

Resistance Training: Exercises aimed at muscle building. Examples include the following: Push-Ups: Classic push-up exercise. Triceps Dips: Bending and extending the arms on a stable surface.

As part of the intervention design, individual goals were established for each adolescent based on their performance during the baseline assessment. The primary objective of this approach was to promote self-referenced progress rather than normative comparisons, thereby fostering intrinsic motivation and supporting long-term engagement in physical activity.

Each participant was encouraged to improve their physical performance by approximately 5–10% relative to their initial measurements. The aims were intended to be both realistic and attainable, while still providing a meaningful challenge to support individual development.

To ensure clarity and enhance motivation, the following message was communicated to all participants at the beginning of the intervention: “Our goal is for each of you to improve compared to your own previous performance. We are not comparing you to others, but to yourselves. Aim to perform better than you did during the first assessment.”

The physical education program was designed flexibly, taking into account the individual’s abilities and fitness level, and if necessary, we modified the training elements. Usually, three training sessions of varying intensity alternated: light, medium, and heavy. Intensity was achieved, for example, by increasing the number of elements. On the fourth occasion, the students could choose their favorite exercises, and in the other half of the training, we held a team game, for example, ball games.

The control group also consisted of high school students who participated in regular physical education classes organized within the school framework, five times a week for 45–60 min each session. Their activities included sports such as gymnastics, running, and ball games such as football, basketball, handball, volleyball, and swimming.

### 2.2. Measurements of Body Weight, Body Mass Index, and Body Fat Percentage

Body weight was measured using an electronic digital scale, recorded to the nearest 0.1 kilogram (kg). The scale was placed on a clean, hard, and level surface. After turning on the scale, students, dressed in light sportswear (without sweatpants) and barefoot, stepped on the scale, and their weight was recorded. Height was measured with a manual stadiometer and recorded to the nearest 0.1 centimeter (cm). Both weight and height were measured once at each measurement occasion, and body mass index (BMI) was calculated.

Body fat percentage was measured using the OMRON BF511 bioelectrical impedance analyzer, which has been validated for assessing body composition in children aged six years and older.

### 2.3. Measurement of Cardiorespiratory Performance

The 20 m shuttle run test was used to measure aerobic capacity. During the test, students were instructed to complete as many 20 m laps as possible, with the running pace dictated by an audio signal. The test was of progressive intensity, starting easy and gradually increasing in difficulty. The audio signal was separated into 21 levels, with the first level allowing 9 s to complete the 20 m distance, decreasing by 1.5 s per level. Students were alerted to change pace by three beeps, indicating they should increase their speed. A lap was considered complete if the student touched or crossed the 20 m line with one foot by the time the beep sounded. If the student reached the line before the beep, they had to wait for the signal before turning back. The test concluded when the participant failed to reach the line at the beep or was unable to continue [24].

After completing the test, we used the following formula to estimate maximum oxygen uptake capacity (VO_2_max): VO_2_max = 0.353 × (number of distances covered) − 1.121 × (age) + 45.619 [25].

### 2.4. Measurement of Muscle Performance

Muscle performance was assessed using five motor tests.

Curl-up Test: Assessed abdominal strength-endurance. Students did sit-ups (1 every 3 s) with bent knees until they reached 75 repetitions or could not continue. Errors were counted by a peer. Final result: total correct sit-ups.

Trunk Lift Test: Measured back muscle strength. Students lifted their trunks, holding a straight head position, while a peer measured the chin-floor distance. The best of two attempts was recorded in cm.

Timed Push-Up Test: Assessed shoulder/arm strength-endurance. Students performed push-ups (1 every 3 s) until reaching 86 repetitions or until failure. Peers counted correct repetitions and noted errors. Final result: total correct push-ups.

Hand Grip Strength: Forearm strength was measured using a dynamometer. Students squeezed the device with maximal effort for 2 s, twice per hand. Final result: best result recorded in kg.

Standing Long Jump Test: Assessed leg dynamic strength. Students performed two jumps, and the better result (heel-to-landing distance) was recorded in cm.

### 2.5. Flexibility Measurement

Flexibility of the lower back and hamstring muscles was assessed using a stable measuring device equipped with a measurement scale. Students performed a maximum forward reach from a sitting position while maintaining the prescribed posture, sliding their hands along the measuring scale on top of the device after three preparatory stretches. The test was repeated for the opposite side by changing leg stance. Results for both sides were recorded to an accuracy of 0.5 cm, and the average of the two values was calculated and recorded in cm. This test differed from the traditional “sit and reach” test in that it measured only one side at a time, allowing for easier detection of side differences.

### 2.6. Data Collection

Measurements and data collection of body composition and cardiorespiratory parameters were performed at three time points: a pretest in May before the intervention began in September, a midpoint assessment in May at the end of the first school year (with no significant differences from baseline, thus not reported), and a posttest in May immediately following the end of intervention.

### 2.7. Statistical Analysis

All statistical analyses were conducted using Prism 9, with a significance level set at *p* < 0.05. Descriptive statistics, including means and standard deviations, were computed for all variables to summarize pre- and post-measurement data.

For longitudinal comparisons within groups between pre- and post-measurements, repeated measures one-way ANOVA was used for variables with normal distributions, accompanied by Šídák’s multiple comparisons test for post hoc analyses. For non-normally distributed variables, Friedman tests were employed, followed by Dunn’s multiple comparisons test to identify specific differences over time. The normality of data distribution was assessed using the Shapiro–Wilk test.

Between-group differences in changes over time were evaluated using independent samples *t*-tests or Mann–Whitney U tests, depending on the data distribution. To investigate predictors of cardiorespiratory performance at post-measurement, a multiple linear regression model was fitted, incorporating BMI, cardiorespiratory performance, and sit-up counts at pre-measurement as independent variables, along with their two-way and three-way interaction terms.

The regression model’s overall fit was evaluated using the coefficient of determination (R^2^), and statistical significance was determined through F-tests and *p*-values. Effect sizes were calculated where relevant to provide additional context for the magnitude of changes observed.

## 3. Results

**Figure 1:**
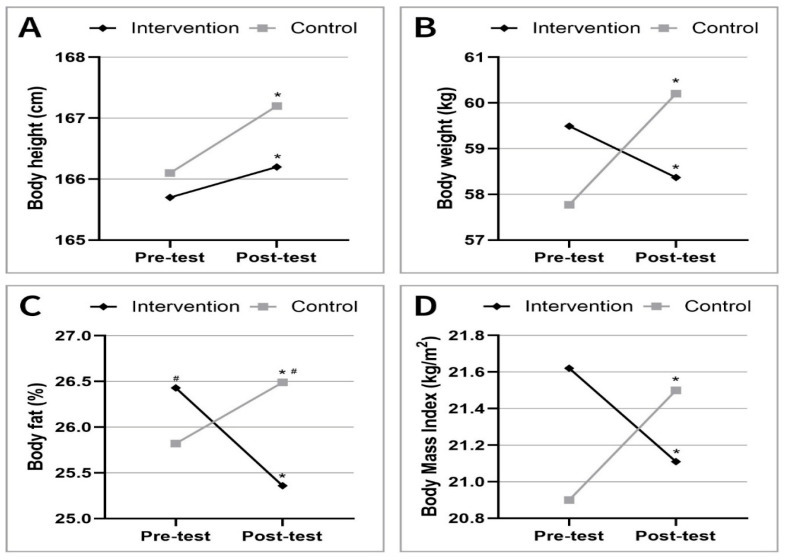
Changes in anthropometric variables ((**A**): Height, (**B**): Weight, (**C**): Body fat percentage, (**D**): Body Mass Index) between pre- and post-measurements for intervention and control groups. Significant differences (^#^ *p* < 0.05; Control vs. Intervention groups) and changes (* *p* < 0.05; pre-test vs. post-test) are indicated.

**Figure 2:**
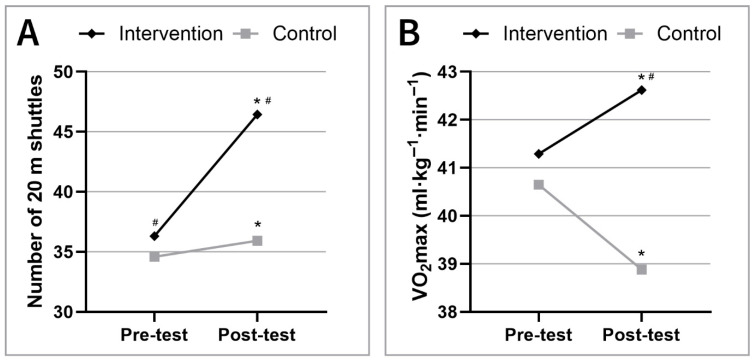
Trends in 20 m shuttle run performance (**A**) and VO_2_max (**B**) over the study period. Significant increase in the cardiorespiratory performance of the intervention group was seen compared to the control group. Significant differences (^#^ *p* < 0.05; Control vs. Intervention groups) and changes (* *p* < 0.05; pre-test vs. post-test) are indicated.

**Figure 3:**
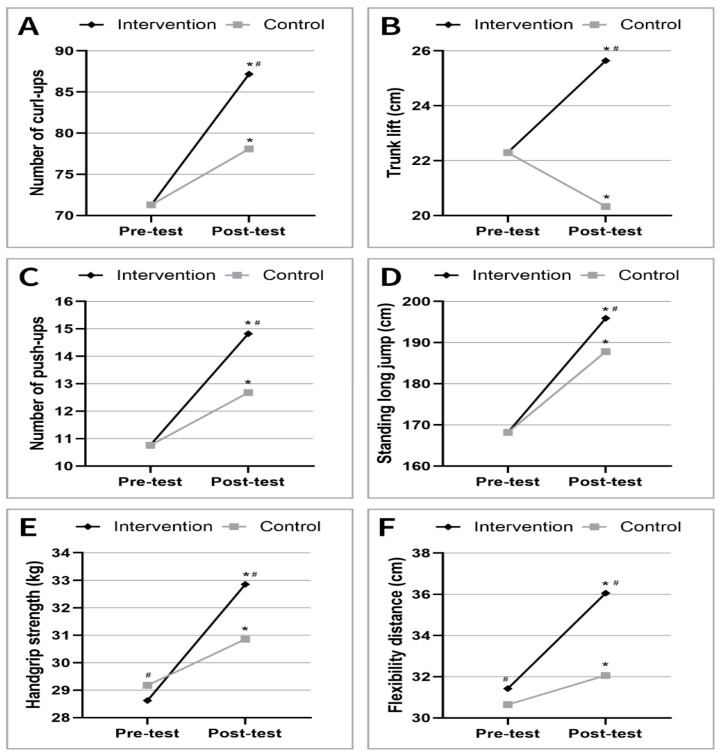
Improvements in muscular strength and flexibility (**A**): Curl-Ups, (**B**): Trunk lift, (**C**): Push-Ups, (**D**): Standing Long Jump, (**E**): Handgrip strength, (**F**): Flexibility) over the study period. The intervention group showed significant improvements in all metrics compared to the control group (* *p* < 0.05 pre-test vs. post-test, ^#^ *p* < 0.05 Control vs. Intervention groups).

Significant changes were observed in anthropometric variables between the pre- and post-measurements in both groups. Figure 1, Figure 2 and Figure 3 illustrate key trends in anthropometric measures, cardiorespiratory fitness, and muscular strength and flexibility, respectively. Body height increased significantly in both the control and intervention groups, with mean changes of +1.1 cm in the control group and +0.5 cm in the intervention group (*p* < 0.05). Body weight also increased in the control group (*p* < 0.05) but decreased slightly in the intervention group during the same period (*p* < 0.05). Notably, BMI exhibited divergent trends, with the intervention group showing a significant reduction from 21.6 ± 3.73 at pre-measurement to 21.1 ± 3.52 at post-measurement (*p* < 0.05), whereas the control group experienced an increase. Similarly, body fat percentage decreased significantly in the intervention group (*p* < 0.05), while the control group showed a modest increase. Comparing the control and intervention groups, the BMI values were not significantly different between the two groups during the study period. Although the control group’s body fat percentage was slightly lower at the beginning of the study, by the end of the study, it had become significantly higher than that of the intervention group (*p* < 0.05) (Figure 1).

In terms of cardiorespiratory performance, the intervention group demonstrated a significant improvement in VO_2_max, with mean values increasing from 41.3 ± 4.84 mL·kg^−1^·min^−1^ to 42.6 ± 5.21 mL·kg^−1^·min^−1^ (*p* < 0.05). In contrast, the control group exhibited a significant decline in VO_2_max, from 40.7 ± 5.49 to 38.9 ± 5.59 mL·kg^−1^·min^−1^ (*p* < 0.05) Comparing the control and intervention groups, VO_2_max values were not significantly different between the two groups at the beginning, but by the end of the study the maximal oxygen uptake was significantly higher in the intervention group (*p* < 0.05) (Figure 2).

Muscular strength and endurance metrics also showed marked improvements in the intervention group. Curl-up performance increased significantly; the number of correct curl-ups changed from 71.3 ± 19.06 to 87.2 ± 18.94 (*p* < 0.05). Similarly, push-up repetitions improved from 10.8 ± 5.77 to 14.8 ± 4.39 at post-measurement (*p* < 0.05). Standing long jump distance increased substantially in the intervention group, from 168.2 ± 27.26 cm to 195.9 ± 25.63 cm (*p* < 0.05). In contrast, the control group exhibited only modest improvements in these metrics, which were not statistically significant. Trunk-lift performance showed divergent trends between the control and intervention groups over the course of the study. In the control group, a slight decline was observed in the mean trunk-lift scores, decreasing from 22.3 ± 5.70 cm at baseline to 20.3 ± 6.60 cm at post-test. In contrast, the intervention group demonstrated a significant improvement in trunk-lift performance, with average scores increasing from 22.3 ± 5.43 cm to 25.6 ± 6.22 cm (*p* < 0.05). This suggests that the intervention had a beneficial effect on lower back and core extensor strength and flexibility, as reflected by the enhanced trunk-lift results. Handgrip strength showed a notable improvement in the intervention group over the course of the study. The mean handgrip strength increased significantly from 28.6 ± 4.19 kg at baseline to 32.9 ± 4.82 kg post-intervention (*p* < 0.05). In contrast, the control group demonstrated only a modest increase in grip strength, from 29.2 ± 4.31 kg to 30.9 ± 4.82 kg.

Comparing the data on muscle strength and endurance measures between the two groups, only the result of the handgrip test was significantly lower at the beginning of the study in the intervention group; the other parameters did not differ from each other. However, by the end of the study, all muscle strength test results were significantly higher in the intervention group compared to the control group (*p* < 0.05).

Flexibility, as assessed by the sit-and-reach test, improved significantly in the intervention group. The mean flexibility increased from 31.4 ± 7.16 cm to 36.1 ± 8.24 cm (*p* < 0.05). While the control group also showed a slight improvement in flexibility, these values remained significantly lower than those of the intervention group by the end of the study period (*p* < 0.05) (Figure 3).

All results were validated using appropriate statistical methods. Repeated measures one-way ANOVA was employed for variables with normal distributions, with Šídák’s multiple comparisons test used for post hoc analyses. For non-normally distributed data, Friedman tests were applied, followed by Dunn’s multiple comparisons test. Statistically significant differences over the study period within groups were indicated as *p* < 0.05. A detailed summary of the results is presented in Table 1.

### Multiple Linear Regression Analysis

A multiple linear regression analysis was performed to identify the predictors of cardiorespiratory performance. The independent variables included BMI, cardiorespiratory performance, and the number of sit-ups completed at the beginning of the study, as well as their two-way and three-way interaction terms.

The model demonstrated a strong overall fit, with an R^2^ = 0.9788, indicating that 97.88% of the variability in cardiorespiratory performance at the end of the study was explained by the predictors. The model was statistically significant (F (7, 79) = 521.1, *p* < 0.0001).

Despite the strong overall fit of the model, none of the individual predictors reached statistical significance at the conventional α = 0.05 threshold. BMI at the beginning had a coefficient of β = 0.5876 (*p* = 0.4686), indicating no significant association with cardiorespiratory performance at the end of the study. Similarly, cardiorespiratory performance at the beginning did not significantly predict cardiorespiratory performance at the end of the study (β = 1.165, *p* = 0.0820). The number of sit-ups at pre-measurement was also not significantly associated with the dependent variable (β = −0.06175, *p* = 0.8069). Furthermore, none of the interaction terms, including BMI x cardiorespiratory performance at the beginning, BMI x sit-ups at the beginning, cardiorespiratory performance x sit-ups at the beginning, or the three-way interaction, were statistically significant (*p* > 0.05).

## 4. Discussion

In our study, we focused on the role of personalized school physical exercise program in improving fitness and body composition in adolescent girls, as it is a well-known fact that physical activity in early life is a determinant of cardiovascular risk factors in both the short and long term [26,27].

In our research, we organized personalized physical exercise classes for adolescent girls twice a week over two school years and compared the results with those of traditional physical education classes. Our results showed that an individualized physical exercise program reduced BMI, body fat percentage, and improved cardiorespiratory performance, muscular strength, and flexibility in adolescent girls, compared to those participating only in traditional physical education classes in a public school.

Physical inactivity is associated with many chronic diseases and places a significant burden on healthcare systems worldwide [26]. Although physical inactivity has become a global phenomenon, most studies have examined adults, when the consequences are already apparent [27]. However, the prevalence of cardiovascular risk factors, including hypertension and obesity, is increasing in adolescents [28]. Nearly one-fifth of adolescents globally are overweight or obese, and the prevalence of adolescent mental disorders (such as depression and anxiety) is also on the rise [1]. Numerous studies have shown that childhood obesity is strongly associated with an increased risk of many chronic diseases in both the short and long term [29]. In our study, the personalized physical exercise program had a beneficial effect on the BMI. Similarly, body fat percentage decreased significantly in the intervention group, while the control group showed a modest increase.

It is known that the responsiveness to fitness training programs is variable. Previous findings in adults have shown considerable heterogeneity in cardiorespiratory fitness (CRF) responses (i.e., measures of VO_2_max) following an exercise intervention [16]. Some studies suggest that the variability in responsiveness may largely be due to the prescribed exercise intensity not accounting for individual metabolic differences. When more traditional approaches to CRF exercise intensity prescription—such as percentages of heart rate reserve—are used, they can potentially lead to the over- or undertraining of participants. A study in adults showed that a personalized program—prescribing exercise based on ventilatory threshold and incorporating functional muscular fitness training—may yield greater training adaptations [30]. In our study, the intervention group demonstrated a significant improvement, while the control group showed a significant decline in cardiorespiratory parameters. Muscular strength also showed marked improvements in the intervention group; in contrast, the control group exhibited only non-significant improvements in these metrics.

Previous research results suggest that early intervention and prevention strategies targeting youth cardiorespiratory fitness might be associated with maintaining health parameters not only in the short term but also later in life [31]. As a short-term benefit, improvements in cardiorespiratory fitness have positive effects on depression, anxiety, mood, and self-esteem, and seem to be associated with higher academic performance [22,32]. As a long-term benefit, evidence suggests that greater cardiorespiratory and muscle performance in adolescents is associated with lower levels of cardiovascular and metabolic risk factors in young adulthood [33,34]. Additionally, higher levels of cardiorespiratory fitness substantially reduce the risk of metabolic syndrome in adulthood, even among those with abdominal obesity during childhood [35,36]. Although long-term conclusions cannot be drawn from our study, the short-term results suggest that improved fitness can be linked to favorable body composition, which is beneficial for cardiovascular health.

There are three key factors that can influence the physical activity of adolescents: the educational setting, the social and digital environment, and the urban environment [1]. Schools are considered one of the fundamental pillars of health promotion worldwide. Several studies suggested that sport opportunities and activities within school settings are positively associated with the overall physical activity levels of adolescent students [37]. The traditional one-size-fits-all teaching methods, however, often struggle to meet the diversity and needs of different students. In education, differentiation creates engaging and challenging experiences that improve engagement and accomplishment by customizing instruction, content, and assessment to match the diverse needs of learners [38].

Affective involvement in physical activity has been gaining increasing attention in efforts to change physical activity behaviors. A randomized controlled trial by Teixeira et al. [15] demonstrated that enjoyable physical activity programs tailored to individual preferences significantly increased participation rates. According to the Affective-Reflective Theory (ART) model, positive emotional experiences not only encourage current participation but also shape future behavior [39]. In our present study, we aimed to accommodate the diversity of the students by tailoring their training plans to their individual interests and athletic abilities to promote their development. In our study, we not only tailored the physical exercise program to the students’ physical ability, but we also took into consideration the actual mood and wishes of the students. The continuous teacher-student communication and a motivating atmosphere were important aspects of every physical education class. According to studies examining motivational processes in physical education, intrinsic motivation toward physical exercise is a significant predictor of adolescents’ daily physical activity [40,41].

### Strengths and Limitations

Our results provide evidence that a personalized physical exercise program, which has received limited academic attention in adolescents, can effectively promote improvements in fitness and body composition parameters when implemented within school physical education classes among adolescent girls. Although the students participated in training for the same duration as in traditional physical education classes, when prescribing the personalized training program, we took into account the students’ initial performance, individual moods and preferences, set individualized and achievable goals, and provided a supportive environment, thereby achieving pronounced improvements in cardiorespiratory performance and body composition parameters. 

It is important to interpret our results in the context of the study’s limitations. First, we only examined the effects of the individualized physical exercise program on physical fitness and body composition parameters; however, it would also be interesting to investigate the effects of affective response, self-efficacy, and autonomous motivation on performance improvement. Secondly, the study was conducted among adolescent girls; therefore, it would be valuable to conduct similar research among adolescent boys as well. Finally, it would be interesting to confirm our results in a larger sample size in future studies.

## 5. Conclusions

This study highlights the importance of a personalized physical exercise program among adolescent girls in achieving high levels of physical performance and promoting a physically active lifestyle, the immediate and long-term health benefits of which are well known. Adolescence is an important period in a person’s life during which significant biological and psychological changes occur within a relatively short period of time. In addition to biological maturation, adolescents also experience increased autonomy in decision-making and develop new lifestyle habits, an important part of which is an appropriate level of physical activity.

Therefore, interventions at this age may have not only short-term effects, but also lifelong beneficial impacts on cardiovascular health.

## Figures and Tables

**Table 1:** Comparison of anthropometric and performance variables between Control and Intervention groups over the study period.

	Control Group		Intervention Group	
	Pre-test	Post-test	Pre-test	Post-test
Body height (cm)	166.1 ± 5.67	167.2 ± 5.88 *	165.7 ± 5.88	166.2 ± 5.48 *
Body weight (kg)	57.8 ± 8.70	60.2 ± 9.60 *	59.5 ± 10.88	58.4 ± 10.21 *
BMI (kg/m^2^)	20.9 ± 2.67	21.5 ± 2.97 *	21.6 ± 3.73	21.1 ± 3.52 *
Body fat (%)	25.8 ± 6.61	26.5 ± 6.21 *#	26.4 ± 6.57	25.4 ± 6.31 *#
20 m shuttles (*n*)	34.6 ± 15.87	35.9 ± 16.13 *#	36.3 ± 13.86	46.4 ± 14.94 *#
VO_2_max (mL·kg^−1^·min^−1^)	40.7 ± 5.49	38.9 ± 5.59 *	41.3 ± 4.84	42.6 ± 5.21 *#
Curl-ups (n)	71.3 ± 19.08	78.1 ± 19.06 *	71.3 ± 19.06	87.2 ± 18.94 *#
Trunk lifts (cm)	22.3 ± 5.70	20.3 ± 6.60 *	22.3 ± 5.43	25.6 ± 6.22 *#
Push-ups (*n*)	10.8 ± 5.72	12.7 ± 4.51 *	10.8 ± 5.77	14.8 ± 4.39 *#
Standing long jumps (cm)	168.2 ± 27.19	187.8 ± 26.40 *	168.2 ± 27.26	195.9 ± 25.63 *#
Handgrip strength (kg)	29.2 ± 4.31	30.9 ± 4.82 *#	28.6 ± 4.19	32.9 ± 4.82 *#
Flexibility (cm)	30.6 ± 7.17	32.1 ± 8.24 *#	31.4 ± 7.16	36.1 ± 8.24 *#

Values are presented as mean ± standard deviation. 20 m shuttles, curl-ups, and push-ups are measured in repetitions (*n*), trunk lifts, and flexibility are measured in centimetres (cm), and handgrip strength is measured in kilograms (kg). Significant differences over the study period within groups are indicated: * *p* < 0.05, differences between Control vs. Intervention groups are indicated: # *p* < 0.05.

## Data Availability

The data presented in this study are available upon request from the corresponding author.
